# *Gadd45g* is required for timely *Sry* expression independently of RSPO1 activity

**DOI:** 10.1530/REP-21-0443

**Published:** 2022-03-22

**Authors:** Nick Warr, Pam Siggers, Joel May, Nicolas Chalon, Madeleine Pope, Sara Wells, Marie-Christine Chaboissier, Andy Greenfield

**Affiliations:** 1 Mammalian Genetics Unit, Medical Research Council, Harwell Institute, Oxfordshire, UK; 2 The Mary Lyon Centre, Medical Research Council, Harwell Institute, Oxfordshire, UK; 3 Université Côte d’Azur, Inserm, CNRS, Institut de Biologie Valrose (iBV), Nice, France

## Abstract

Sex determination in mammals is controlled by the dominance of either pro-testis (SRY-SOX9-FGF9) or pro-ovary (RSPO1-WNT4-FOXL2) genetic pathways during early gonad development in XY and XX embryos, respectively. We have previously shown that early, robust expression of mouse *Sry* is dependent on the nuclear protein GADD45g. In the absence of GADD45g, XY gonadal sex reversal occurs, associated with a major reduction of *Sry* levels at 11.5 dpc. Here, we probe the relationship between *Gadd45g* and *Sry* further, using gain- and loss-of-function genetics. First, we show that transgenic *Gadd45g* overexpression can elevate *Sry* expression levels at 11.5 dpc in the B6.Y^POS^ model of sex reversal, resulting in phenotypic rescue. We then show that the zygosity of pro-ovarian *Rspo1* is critical for the degree of gonadal sex reversal observed in both B6.Y^POS^ and *Gadd45g*-deficient XY gonads, in contrast to that of *Foxl2*. Phenotypic rescue of sex reversal is observed in XY gonads lacking both *Gadd45g* and *Rspo1*, but this is not associated with rescue of *Sry* expression levels at 11.5 dpc. Instead, *Sox9* levels are rescued by around 12.5 dpc. We conclude that *Gadd45g* is absolutely required for timely expression of *Sry* in XY gonads, independently of RSPO1-mediated WNT signalling, and discuss these data in light of our understanding of antagonistic interactions between the pro-testis and pro-ovary pathways.

## Introduction

Mammalian sexual development involves the commitment of the bipotential gonadal primordium to a testicular or ovarian fate (Greenfield 2013). In mice, testis determination is dependent on the timely expression of the Y-linked gene *Sry* in supporting cell precursors of the developing XY gonad (Bullejos & Koopman 2001). SRY protein, a transcription factor, acts through regulatory elements of the *Sox9* gene in order to effect its up-regulation (Sekido & Lovell-Badge 2008, Gonen *et al.* 2017, 2018). Subsequently, SOX9, another transcription factor, binds target genes in order to direct the differentiation of Sertoli cells, which orchestrate morphogenetic processes required for testis development (Cool *et al.* 2012, Rahmoun *et al.* 2017). In XX gonads, the absence of SRY leads to distinct pro-ovarian pathways of gene expression gaining dominance; these comprise canonical WNT signalling, especially RSPO1- and WNT4-dependent signals, and the activity of the pioneer transcription factor FOXL2 (Pannetier *et al.* 2016).

The establishment of such sexually dimorphic pathways of gene expression is dependent on the mutually antagonistic interactions between them in early gonad development (Greenfield 2015). Any significant reduction or delay in *Sry* expression during a critical phase of testis determination can result in XY gonadal sex reversal (Hiramatsu *et al.* 2009, Carre *et al.* 2018), due to a failure of such inhibition. Moreover, mutually antagonistic molecular interactions exist at the heart of the sex-determining mechanism: the ovarian determinant RSPO1 inhibits the activity of the testis-determining factor ZNRF3, which itself inhibits canonical WNT signalling required for ovary development (Harris *et al.* 2018).

A number of gene mutations have been reported that disrupt *Sry* regulators and these can cause testis determination phenotypes (reviewed in Carre & Greenfield 2014, Larney *et al.* 2014, Nef *et al.* 2019). While such disrupted genes were assumed to act directly on *Sry* expression in a positive manner, it has recently been shown that one inhibitor of the pro-ovarian canonical WNT signalling pathway is required for the establishment of robust *Sry* expression (Garcia-Moreno *et al.* 2019). CBX2, a component of the polycomb repressive complex1 that binds H3K27me3 to mediate gene silencing, is a known factor required for normal *Sry* expression (Katoh-Fukui *et al.* 2012). However, *Sry* expression is re-established in fetal XY gonads of embryos lacking both CBX2 and WNT4, suggesting that CBX2 acts primarily in a negative fashion to oppose the influence of WNT4/beta-catenin (Garcia-Moreno *et al.* 2019). Moreover, these data suggest that elimination of pro-ovarian WNT4/beta-catenin signals in the early XY gonad is a prerequisite of establishing a molecular environment in which early, robust *Sry* expression can occur.

We have previously reported a series of loss- and gain-of-function studies that reveal a role for the mitogen-activated protein kinase (MAPK) signalling pathway in testis determination via its effect on *Sry* expression. Loss of the kinase MAP3K4, or its regulator GADD45g, results in embryonic XY gonadal sex reversal associated with reduced *Sry* expression around 11.5 dpc on the C57BL/6J (B6/J) mouse strain (Bogani *et al.* 2009, Warr *et al.* 2012). Spatiotemporal profiling suggests that *Sry* expression eventually recovers to near-normal levels in both *Map3k4*- and *Gadd45g*-deficient XY gonads, but too late to affect the developmental fate of gonadal somatic cells, which acquire an ovarian fate as a consequence (Warr *et al.* 2012). Moreover, overexpression of *Map3k4* from a functional BAC transgene has been shown to rescue an *Sry* deficit in the classical mouse sex reversal mutation *T*-associated sex reversal (*Tas*) by re-establishing robust levels of *Sry* (Warr *et al.* 2012, 2014).

Here, we extend our analysis of the role of GADD45g in sex determination by probing its relationship with *Sry* further, using loss- and gain-of-function mouse lines. Using a previously reported BAC transgene (Warr *et al.* 2018), we show that transgenic overexpression of *Gadd45g* rescues the B6.Y*^POS^* model of XY gonadal sex reversal by promoting *Sry^POS^* expression. Exploiting this transgenic rescue, we then generated B6.Y*^POS^* fetuses that lack one copy of either *Rspo1*or *Foxl2* in order to assess the relative contribution of these two main pro-ovarian genes to XY fetal ovary development. B6.Y*^POS^*, *Rspo1*^−/+^ heterozygous fetuses at 14.5 dpc exhibit substantial rescue of gonadal sex reversal defects, with testicular tissue forming throughout much of the gonad. By contrast, B6.Y*^POS^*, *Foxl2*^−/+^ heterozygotes, as well as homozygotes, show only minimal evidence of testicular tissue development. We then assessed whether loss of *Rspo1* operates to rescue sex reversal by rescuing *Sry* expression. We show that gonads in XY fetuses lacking both *Gadd45g* and *Rspo1*develop as ovotestes with substantial testicular tissue formation at 14.5 dpc. However, this rescue of gonadal sex reversal in the double knockout is not associated with any rescue of the levels of *Sry* expression at 11.5 dpc, with only minimal *Sry* expression detected, as in XY *Gadd45g*-deficient gonads. We conclude that GADD45g is absolutely required for timely *Sry* expression independently of RSPO1 activity and that, therefore, loss of RSPO1 promotes testis determination in the absence of GADD45g through positive impacts downstream of initiation of timely *Sry* expression.

## Materials and methods

### Mouse strains and breeding

Breeding of B6.Y*^POS^* mice has been previously described (Livermore *et al.* 2020). BAC transgenic lines harbouring functional *Map3k4* and *Gadd45g* have been previously described (Warr *et al.* 2014, 2018). *Gadd45g* knockout (KO) mice, *Foxl2* KO mice (a kind gift from Robin Lovell-Badge and Mathias Treier) and *Rspo1*KO mice have also previously been described (Hoffmeyer *et al.* 2001, Schmidt *et al.* 2004, Chassot *et al.* 2008). Mice were bred and maintained on the C57BL/6J background. All animal experiments were approved by the Animal Welfare and Ethical Review Body at MRC Harwell. Mice used were bred under a license from the UK Home Office (PPL 70/8898). Mice were housed in individually ventilated cages in a specific pathogen-free environment. Further details of micro- and macroenvironmental conditions are available on request.

### Generation of embryos

Noon on the day of the copulatory plug was counted as 0.5 days *post coitum* (dpc). Embryos collected at 11.5dpc were staged accurately based on the number of tail somites (ts).

### Whole-mount *in situ*hybridization

Whole-mount *in situ*hybridization (WMISH) analysis of embryonic tissues was performed as previously described (Grimmond *et al.* 2000, Cox *et al.* 2006). Probes for *Sox9* (Wright *et al.* 1995) and *Stra8* (Bogani *et al.* 2009, Warr *et al.* 2012), *Sry* (Bullejos & Koopman 2001) and *3b-Hsd* (Siggers *et al.* 2002) have been previously described.

### Immunostaining

Gonads were fixed overnight in 4% PFA and processed for wax sectioning. Sections were dewaxed in xylene and antigen retrieval was achieved using Declere solution (Cell Marque, Rocklin, CA, USA). The following primary (1:100) and secondary (1:200) antibodies were used: anti-DDX (Abcam ab13840) and anti-AMH (Santa Cruz sc6886), donkey-anti-rabbit Alexa Fluor 594 (Invitrogen A21207) and donkey-anti-goat Alexa Fluor 488 (Invitrogen A11055). Images were captured using a Zeiss LSM 710 upright confocal microscope and Zen software.

### Quantitative reverse transcription-PCR

RNA was extracted using RNAeasy micro kit (Qiagen,74004) according to the manufacturer’s instructions. cDNA was synthesized from approximately 100 ng total RNA (one pair of sub-dissected gonads) using random primers (high capacity cDNA RT kit, Applied Biosystems, 4368814). Relative cDNA levels were analysed by quantitative PCR using an Applied Biosystems 7500 real-time PCR machine. The TaqMan-MGB-FAM gene expression assays (Applied Biosystems) used were: *Rps29* (Mm02342448_gH) and *Sry* (Mm00441712_s1).

## Results

**Figure 1:**
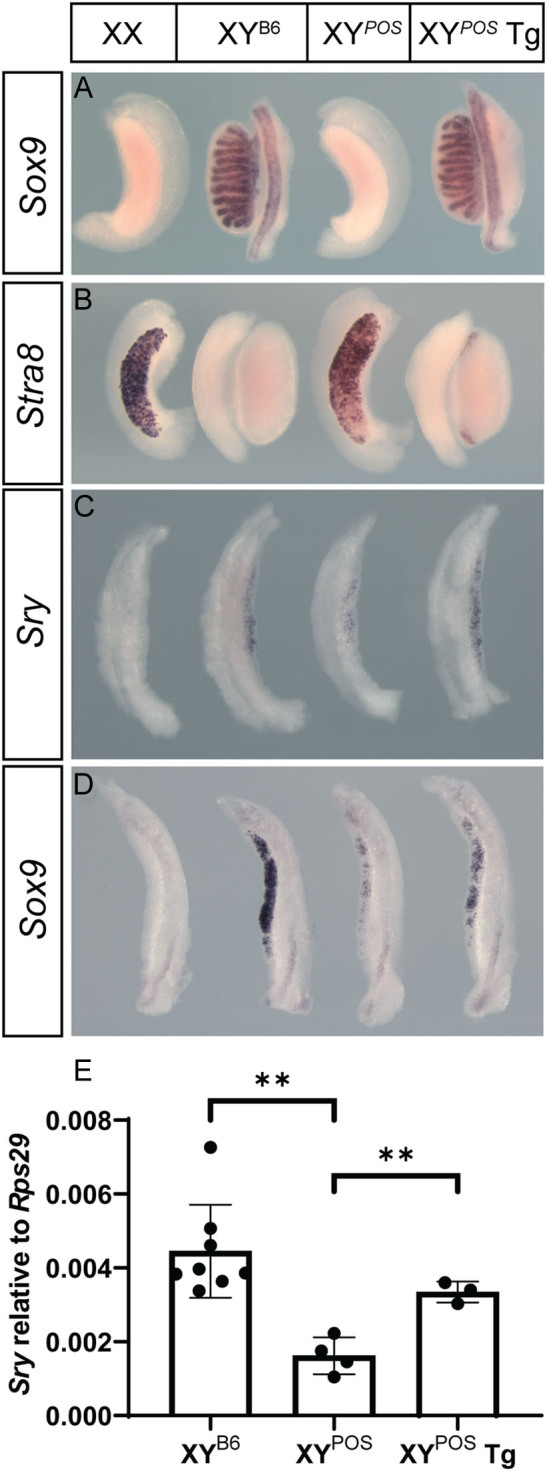
Rescue of fetal B6.Y^POS^ gonadal sex reversal by BAC transgenic overexpression of *Gadd45g* through positive impact on *Sry*. Whole-mount *in situ* hybridisation (WMISH) of XX wild-type, XY (B6.Y^B6^) wildtype, B6.Y^POS^ and transgenic B6.Y^POS^ gonads (left to right) at 14.5 dpc with *Sox9* (A) and *Stra8* (B) probes. WMISH analysis of fetal gonads with the same genotypes at 11.5 dpc (16 ts, *Sry*, C) and *Sox9* (19ts, D). Quantitative reverse transcription-PCR analysis of *Sry* expression (E) confirms WMISH data, with significant reduction in *Sry* levels in B6.XY^POS^ (compared to B6.XY^B6^ controls) being rescued to near-control levels by transgene expression (B6.XY^POS^ Tg). **P ≤ 0.01 (two-tailed* t-*test); B6 = C57BL6/J.

We have shown previously that a bacterial artificial chromosome (BAC) encoding *Gadd45g* is capable of rescuing sex determination defects in a *Gadd45g* knockout, indicating its functionality (Warr *et al.* 2018). We first sought to assess the impact of *Gadd45g* overexpression in a classical model of XY gonadal sex reversal, the B6.Y*^POS^* mouse (Eicher *et al.* 1982), by crossing the *Gadd45g* BAC transgene onto this strain. Testis determination defects in B6.Y*^POS^* are associated with dysregulation of *Sry* expression (Bullejos & Koopman 2005, Livermore *et al.* 2020), presumably due to some functional mismatch between the *Sry^POS^* allele and autosomal/X-linked gene variants in B6/J mice. We recently reported an autosomal modifier that protects against B6.Y*^POS^* sex reversal (Livermore *et al.* 2020): all experiments described here used mice that lack this modifier. We first examined B6.Y*^POS^* transgenic gonads at 14.5 dpc using WMISH with *Sox9* and *Stra8* (Fig. 1). Control B6.Y*^POS^* gonads were most commonly ovaries that lacked the Sertoli cell marker *Sox9* (Fig. 1A). However, all transgenic gonads had a testicular morphology, with testis cords extending almost to the poles of the gonad, like B6.Y^B6^ controls. Some polar expressions of *Stra8*, a marker of germ cell meiotic entry that is strongly expressed in XX and B6.Y*^POS^* controls, in transgenic gonads indicates that rescue of sex reversal is not absolutely complete (Fig. 1B). However, this does not prevent the development of fertile males in B6.Y*^POS^*, *Gadd45g* BAC transgenic mice. Such males were routinely bred to B6 females to generate breeding stock and embryos for experimentation. When a functional BAC transgene encoding the kinase *Map3k4*(Warr *et al.* 2014), which interacts with *Gadd45g* in testis determination (Warr *et al.* 2012), was bred onto B6.Y*^POS^*, the same rescue of fetal gonadal sex reversal was observed (Supplementary Fig. 1, see section on supplementary materials given at the end of this article). But our focus here is the role of *Gadd45g*.

We then carefully examined the expression of *Sry* and *Sox9* at 11.5 dpc, the sex-determining stage of gonad development, in control and *Gadd45g* transgenic B6.Y*^POS^* gonads (Fig. 1C, D and E). We confirmed that *Sry* expression was reduced at the 16 tail somite (ts) stage in B6.Y*^POS^* gonads when compared to B6.Y^B6^ controls (Fig. 1C), consistent with earlier reports. Similarly, and presumably as a consequence, *Sox9* expression was greatly reduced at these early stages (Fig. 1D). However, in the presence of the *Gadd45g* BAC transgene, *Sry* expression levels were higher, compared with those detected in B6.Y^B6^ controls (Fig. 1C). These semi-quantitative WMISH data on *Sry* expression were confirmed by quantitative RT-PCR analysis, suggesting that WMISH is a reliable method for detecting its expression (Fig. 1E). *Sox9* expression also recovered, though not to levels comparable to B6.Y^B6^ controls, perhaps due to the presence of the SRY^POS^ protein isoform (Fig. 1D). These data suggest that overexpression of *Gadd45g* can have a positive impact on *Sry^POS^* expression. However, they do not reveal whether this positive impact reflects an absolute requirement for GADD45g in timely expression of *Sry*, or whether, like CBX2 (Garcia-Moreno *et al.* 2019), this requirement is dependent on levels of canonical WNT signalling.

**Figure 2:**
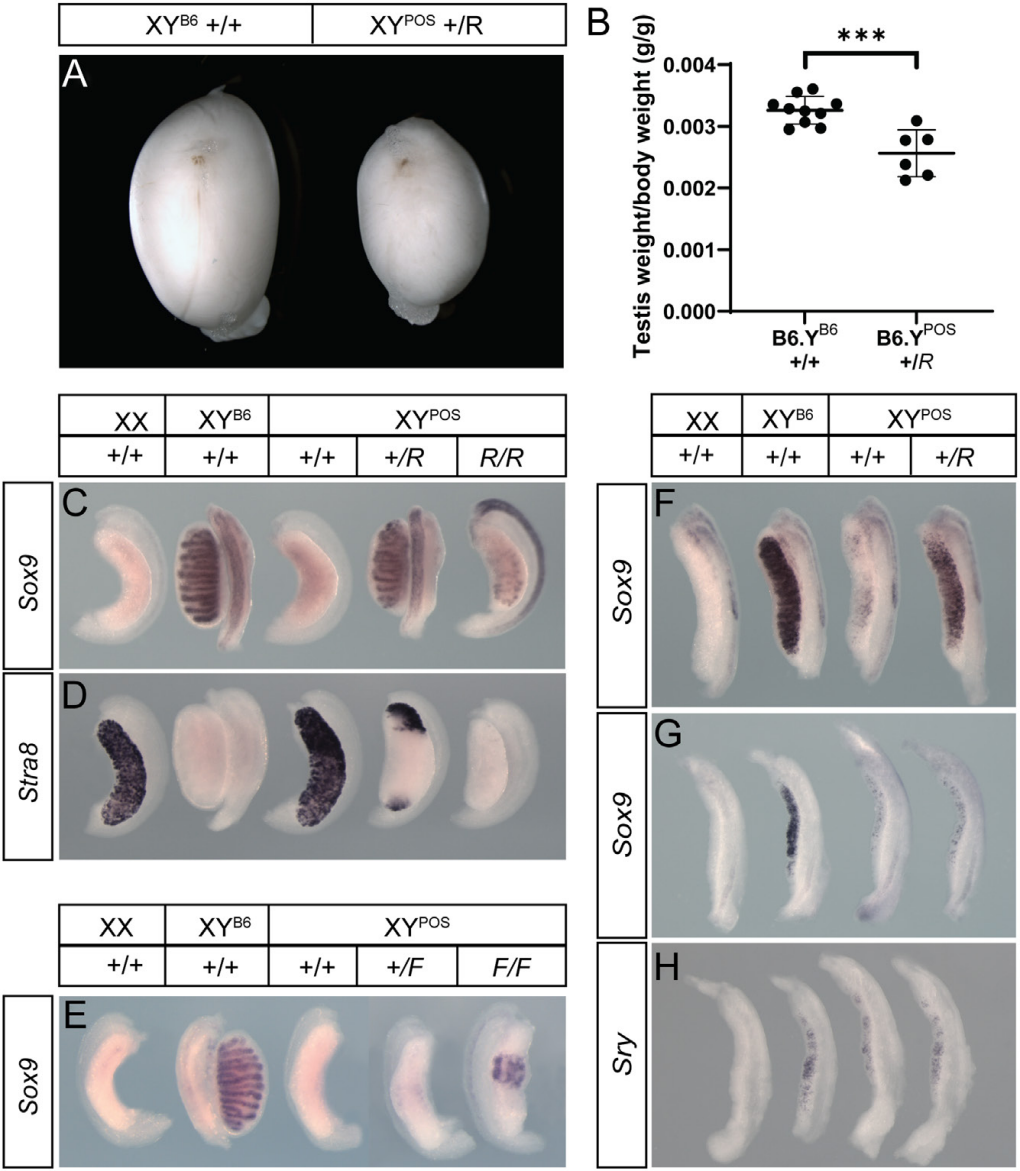
Rescue of B6.Y^POS^ gonadal sex reversal by genetic ablation of *Rspo1*. (A) Images of adult testes (11.5 weeks) from control (XY^B6^) and B6.Y^POS^, *Rspo1*^+/−^ heterozygous (+/R) animals; (B) Testis weights of the animals from the same genotypic cohorts (per terminal body weight). WMISH of XX WT, XY (B6.Y^B6^) WT, B6.Y^POS^ and B6.Y^POS^ embryos lacking one (+/*R*) or two (*R/R*) copies of *Rspo1* (left to right) at 14.5 dpc with *Sox9* (C) and *Stra8* (D) probes; WMISH of XX WT, XY (B6.Y^B6^) WT, B6.Y^POS^ and B6.Y^POS^ embryos lacking one (+/*F*) or two (*F/F*) copies of *Foxl2* (left to right) at 14.5 dpc with *Sox9* probe (E); WMISH of XX WT, XY (B6.Y^B6^) WT, B6.Y^POS^ and B6.Y^POS^ embryos lacking one copy (+/*R1*) of *Rspo1* (left to right) with *Sox9* probe at 12.5 dpc (F) and 11.5 dpc (G); and with an *Sry* probe at 11.5 dpc (H). ***P ≤ 0.001 (two-tailed *t*-test); B6 = C57BL6/J.

To test the role of canonical WNT signalling in the context of testis determination defects on B6/J, we used a loss-of-function *Rspo1* allele (Chassot *et al.* 2008) to assess its impact on B6.Y*^POS^* gonadal sex reversal. As a control, we also examined the impact of a *Foxl2* null allele (Schmidt *et al.* 2004), thereby assessing the role of the two major pro-ovarian (granulosa cell) pathways in XY fetal ovary development. B6.Y*^POS^* adults lacking a single copy of *Rspo1* (*Rspo1^−/+^*) were scored as males at weaning and at necropsy (12 weeks of age) were found to contain testes of reduced size compared to B6.Y^B6^ controls, but with no overt changes in morphology or anatomy (Fig. 2A, B and Supplementary Fig. 2). Examination of fetal gonads at 14.5 dpc in B6.Y*^POS^, Rspo1^−/+^* animals by WMISH revealed substantial rescue of gonadal sex reversal, with extensive *Sox9*-positive testis cord formation throughout much of the gonad (Fig. 2C), which lacked *Stra8* expression apart from at the poles (Fig. 2D). B6.Y*^POS^, Rspo1^−/−^* homozygous fetal gonads at 14.5 dpc also had testis cords and completely lacked *Stra8* expression, but *Sox9* expression was reduced and the testes were smaller than B6.Y*^POS^, Rspo1^−/+^* counterparts (Fig. 2C and D). Control gonads (B6.Y*^POS^, Rspo1^+/+^*) were uniformly ovarian by contrast (Fig. 2C and D). Equivalent examination of B6.Y*^POS^, Foxl2^−/+^* fetal gonads 14.5 dpc revealed no significant rescue of XY gonadal sex reversal, with only very modest levels of *Sox9*-positive testicular tissue (Fig. 2E). The rescue was a little more pronounced in B6.Y*^POS^, Foxl2^−/−^* gonads, with a small amount of testicular tissue forming centrally, but still not to the extent of B6.Y*^POS^, Rspo1^−/+^* gonads (Fig. 2E). From this, we conclude that RSPO1-mediated signalling, and canonical WNT signalling by implication, is the dominant regulator of granulosa cell fate specification during B6.Y*^POS^* sex reversal, and XY ovary development more generally. By comparison, FOXL2 plays a relatively minor role.

We then examined rescue of gonadal sex reversal in B6.Y*^POS^, Rspo1^−/+^* fetuses at earlier stages. Examination of *Sox9* at 12.5 dpc revealed robust expression throughout the gonad, in contrast to B6.Y*^POS^, Rspo1^+/+^* controls, although without significant testis cord formation, suggesting a delay in testis determination (Fig. 2F). However, neither *Sox9* (Fig. 2G) nor *Sry* (Fig. 2H) were up-regulated at 11.5 dpc in B6.Y*^POS^, Rspo1^−/+^* fetuses relative to in B6.Y*^POS^, Rspo1^+/+^* controls. This suggests that the mechanism of rescue caused by haploinsufficiency of *Rspo1* differs from that caused by transgenic overexpression of *Gadd45g*(compare with Fig. 1).

**Figure 3:**
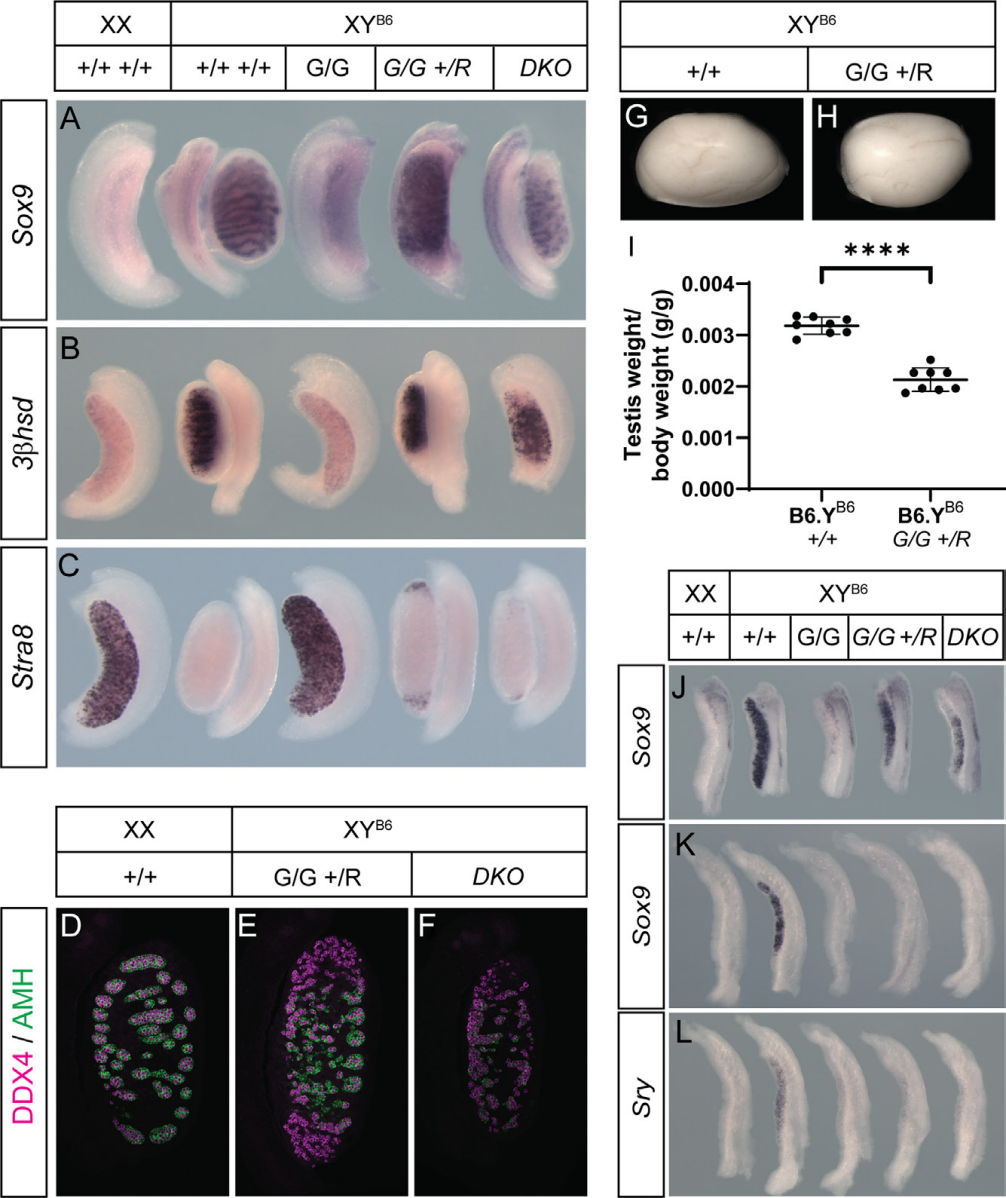
Rescue of gonadal sex reversal in mice lacking *Gadd45g* by genetic ablation of *Rspo1*. WMISH of XX and XY^B6^ WT (+/+), XY *Gadd45g*^−/−^ (*G*/*G*), XY *Gadd45g*^−/−^, *Rspo1*^+/−^ (*G*/*G*, +/*R*) and XY *Gadd45g*^−/−^, *Rspo1*^−/−^ (DKO) fetal gonads at 14.5 dpc with *Sox9* (A), Leydig cell marker *3b-Hsd* (B) and *Stra8* (C) probes; immunostaining of XY^B6^ +/+ (D), *G*/*G*, +/*R* (E) and DKO (F) gonadal sections at 14.5 dpc with anti-DDX4 (magenta) and anti-AMH (green) antibodies; adult testes (10 weeks) from XY +/+ (G) and XY *G*/*G*, +/*R* (H) males with weights (per terminal body weight) (I); WMISH of XX and XY^B6^ +/+, XY *G*/*G*, XY *G*/*G*, +/*R* and XY DKO fetal gonads with *Sox9* at 12.5 (J) and 11.5 dpc (K) and *Sry* at 11.5 dpc (17 ts) (L). ****P ≤ 0.0001 (two-tailed *t*-test); B6 = C57BL6/J.

We then exploited this role for *Rspo1* in XY ovary development by testing whether GADD45g regulates *Sry* expression in an RSPO1-dependent fashion by generating *Gadd45g* null mice (*Gadd45g*^−/−^) that also lacked one or two copies of *Rspo1*. We first examined XY gonad development at 14.5 dpc in *Gadd45g*^−/−^,* Rspo1*^−/+^ (GhomRhet) and *Gadd45g*^−/−^,* Rspo1*^−/−^ (double knockout, DKO) fetuses, and controls (Fig. 3A, B, C, D, E and F). Examination of Sertoli cell (Fig. 3A), Leydig cell (Fig. 3B) and meiotic germ cell (Fig. 3C) development using cell lineage markers revealed a substantial degree of rescue of sex in both XY GhomRhet and DKO fetal gonads. Both types of rescued gonads exhibited *Sox9*- and AMH-positive Sertoli cells and DDX4-positive germ cells in reasonably well-formed testis cords throughout much of the organ (Fig. 3D, E and F), although this was variable from mutant to mutant. Rescue was generally more complete in XY DKO gonads; however, as with B6.Y*^POS^, Rspo1^−/−^* gonads, these were smaller than GhomRhet gonads (Fig. 3). XY *Gadd45g*^−/−^ gonads at the same stage were ovaries with no *Sox9* expression or Leydig cells (*3b-Hsd*-negative), but exhibiting robust *Stra8* express (Fig. 3A, B and C). In contrast to XY *Gadd45g*^−/−^ adult mice, which are phenotypic females, XY GhomRhet mice were fully masculinized males with hypoplastic testes (Fig. 3G, H, I and Supplementary Fig. 2).

Finally, we examined *Sox9* and *Sry* expression in control, GhomRhet and XY DKO fetal gonads (Fig. 3J, K and L). At 12.5 dpc, *Sox9* expression was robust in GhomRhet and XY DKO gonads compared to *Gadd45g*^−^^/−^ controls (Fig. 3J). However, at 11.5 dpc (19 ts), no *Sox9* expression was detectable in any gonads lacking *Gadd45g* following standard WMISH (Fig. 3K) and *Sry* expression was also minimal at 11.5 dpc (17 ts) in the same mutant gonads, compared to robust expression in B6.Y^B6^ control gonads at this stage (Fig. 3L). We conclude that timely *Sry* expression requires GADD45g function irrespective of the zygosity, and thus levels of expression, of *Rspo1*.

## Discussion

The study reported here further underlines the intimate positive relationship between *Gadd45g* and *Sry* expression and function. We have previously shown that the temporal expression profile of each gene is remarkably similar, with initiation in the centre of the developing gonad at around 10.5 dpc, prior to overt gonad differentiation, and cessation of expression once this is completed, at 13.5 dpc (Warr *et al.* 2012). The regulation of *Sry* expression by GADD45g is suggested by these profiles, especially given that expression of the latter precedes that of the former (Warr *et al.* 2012). Moreover, the expression of both is restricted to supporting cell precursors (Warr *et al.* 2012, Stevant *et al.* 2019). The spatiotemporal expression profile of these genes is, therefore, indicative of a role in testis determination, now clearly established for *Gadd45g* by a number of genetic ablation studies (Gierl *et al.* 2012, Warr *et al.* 2012, Johnen *et al.* 2013). We and others have reported a role for GADD5g in activation of MAPK signalling, in sex determination (Gierl *et al.* 2012, Warr *et al.* 2012) and other contexts (Lu *et al.* 2001). However, additional molecular functions cannot be excluded: for example, other members of the GADD45 family are implicated in epigenomic functions on chromatin (Barreto *et al.* 2007, Schmitz *et al.* 2009, Niehrs & Schafer 2012, Schafer *et al.* 2013), so much remains to be elucidated concerning the role of GADD5g in sex determination.

We report testicular development in fetal XY gonads lacking both *Gadd45g* and *Rspo1* (XY DKO), i.e. rescue of XY gonadal sex reversal in *Gadd45g*-deficient fetal gonads by genetic ablation of *Rspo1*, in contrast to the essentially ovarian development of XY gonads lacking both *Gadd45g* and *Foxl2* (see Supplementary Fig. 3). This indicates that *Rspo1*, and downstream canonical WNT signals, are the primary determinant of ovarian tissue development in an XY *Gadd45g*-deficient context. The relatively insignificant role for *Foxl2* in this genetic context may reflect the later expression of this gene during fetal gonad development in mice (Auguste *et al.* 2011, Niu & Spradling 2020). The absence of any significant increase in *Sry* expression levels in XY DKO gonads at 11.5 dpc, when compared to XY *Gadd45g*-deficient gonads, indicates an absolute requirement for GADD45g in directing early, robust expression of *Sry*, even in the absence of the pro-WNT/pro-ovarian gene *Rspo1*. This observation is in contrast to the situation reported for XY gonads lacking both *Cbx2*and *Wnt4* (Garcia-Moreno *et al.* 2019). CBX2, also required for normal *Sry* expression (Katoh-Fukui *et al.* 2012), acts by epigenomic inhibition of WNT gene expression and in its absence XY gonadal sex reversal is observed. Rescue of sex reversal is observed upon additional genetic ablation of *Wnt4*, but rescue in *Cbx2^-/-^, Wnt4^-/-^* XY DKO gonads is associated with restoration of *Sry* expression levels at 11.5 dpc (Garcia-Moreno *et al.* 2019). It is unclear why similar rescue of *Sry* expression is not observed in the XY DKO reported here. It may reflect differences in background strain or *Sry* detection methods; alternatively, it may reflect differing impacts on canonical WNT signalling caused by loss of *Rspo1* and *Wnt4*, or, more likely, an essential and positive role for GADD45g in *Sry* regulation not replicated by the essentially negative, inhibitory role of CBX2 in opposing WNT signalling.

It is also interesting to note that significant rescue of gonadal sex reversal due to the absence of *Gadd45g* occurs when one or two functional copies of *Rspo1* are removed, suggesting that dosage of this pro-WNT gene is important in XY fetal ovary development. This is notable given that ablation of *Rspo1* does not significantly disrupt fetal XX ovary development, i.e. does not result in the appearance of testicular tissue in the fetal gonad, as is also the case following the loss of either *Wnt4* or *Foxl2* (Pannetier *et al.* 2016). This is presumably due to the absence of *Sry* in the XX context and/or functional redundancy with other pro-ovarian (anti-testis) factors. Indeed, partial rescue of XY fetal testis development in other examples of a double knockout involving a pro-testis and pro-ovarian gene pair (Lavery *et al.* 2012, Nicol & Yao 2015, Garcia-Moreno *et al.* 2019) is likely to be due to the presence of *Sry*. It is our conclusion that in the absence of *Gadd45g* and pro-WNT *Rspo1*, a testis-determining gene, perhaps *Sox9* or another pro-testis gene (Nicol & Yao 2015, Richardson *et al.* 2020), can *eventually* respond satisfactorily to SRY even when there is a delay in *Sry* reaching functional levels. It is also worth noting that loss of both copies of *Rspo1* results in an XY DKO (XY *Gadd45g*^-/-^,* Rspo1*^-/-^) gonad with testicular morphology of reduced size and with less prominent *Sox9* expression than the XY GhomRhet (*Gadd45g*^-/-^,* Rspo1*^-/+^) counterpart. This presumably reflects the role of RSPO1 and WNT signalling in cell proliferation in the early gonad (Jeays-Ward *et al.* 2004). The impact on cell proliferation caused by loss of *Rspo1* results in the development of hypoplastic XY gonads with a reduced number of Sertoli cells (Gregoire *et al.* 2018). This impact of the loss of RSPO1 on Sertoli cell differentiation may also explain the less robust formation of testis cords, and associated reduced *Sox9* expression, in B6.Y*^POS^* testes lacking *Rspo1* when compared to their heterozygous counterparts (see Fig. 2C).

In conclusion, we show that the requirement for GADD45g to initiate timely expression of *Sry* during testis determination is not contingent on the presence of RSPO1-dependent signals i.e. WNT signals. This observation is consistent with the model involving an essential role for GADD45g in direct activation of *Sry* transcription through MAPK signals (Gierl *et al.* 2012, Warr *et al.* 2012), but it does not rule out additional, perhaps negative, roles for *Gadd45g* in testis determination. The fact that rescue of B6.Y^POS^ sex reversal is more complete when *Gadd45g* is overexpressed by transgenesis, compared to the loss of *Rspo1*, possibly reflects other roles for GADD45g in promoting pro-testis gene expression or opposing pro-ovary genes.

## Supplementary Material

Figure S1. Rescue of fetal B6.Y<sup>POS</sup> gonadal sex reversal by BAC transgenic overexpression of Map3k4. Wholemount in situ hybridisation (WMISH) of (left to right) B6.XX wild-type, B6.XY (XY<sup>B6</sup>) wildtype, B6.XY<sup>POS</sup> and transgenic B6.XY<sup>POS</sup> (M3K4 Tg) gonads at 14.5 dpc with Sox9 (A) and Stra8 (B) probes. B6 = C57BL6/J

Figure S2. Histological examination of adult testis sections. A) control XY (B6.XY<sup>B6</sup> +/+), B) B6.XY<sup>POS</sup> +/Rspo1 heterozygous (+/R) and C) compound B6.XY<sup>B6</sup> homozygous Gadd45g/Gadd45g, heterozygous +/Rspo1 (G/G, +/R) mice reveal no overt differences in anatomy. B6 = C57BL6/J; Scale bar = 100 µm.

Figure S3. XY fetal gonads lacking both Gadd45g and Foxl2 have an ovarian morphology. Comparative Sox9 WMISH analysis of (left to right) B6 control XX and XY, homozygous XY Gadd45g/Gadd45g (G45g/G45g) and doubly homozygous XY Gadd45g/Gadd45g, Foxl2/Foxl2 mutant (G45g:Foxl2 DKO) gonads at 14.5 dpc. One of the two XY DKO gonads (far right) exhibits low levels of Sox9 in the centre of the ovary. B6 = C57BL6/J

## Declaration of interest

The authors declare that there is no conflict of interest that could be perceived as prejudicing the impartiality of the research reported.

## Funding

This research was supported by support to A G (MC_U142684167) through core funding at the Mammalian Genetics Unit, MRC Harwell Institute.

## Author contribution statement

N W, P S, N C, J M, M P and S W performed the study and analysed data. M C and A G conceived the study. A G wrote the manuscript.
