# Supplementary figures and images for: Gadd45g is required for timely Sry expression independently of RSPO1 activity
Source: Reproduction. 2022 Mar 22;163(6):333–40. doi: 10.1530/REP-21-0443 (PMC9066659; doi:10.1530/REP-21-0443)

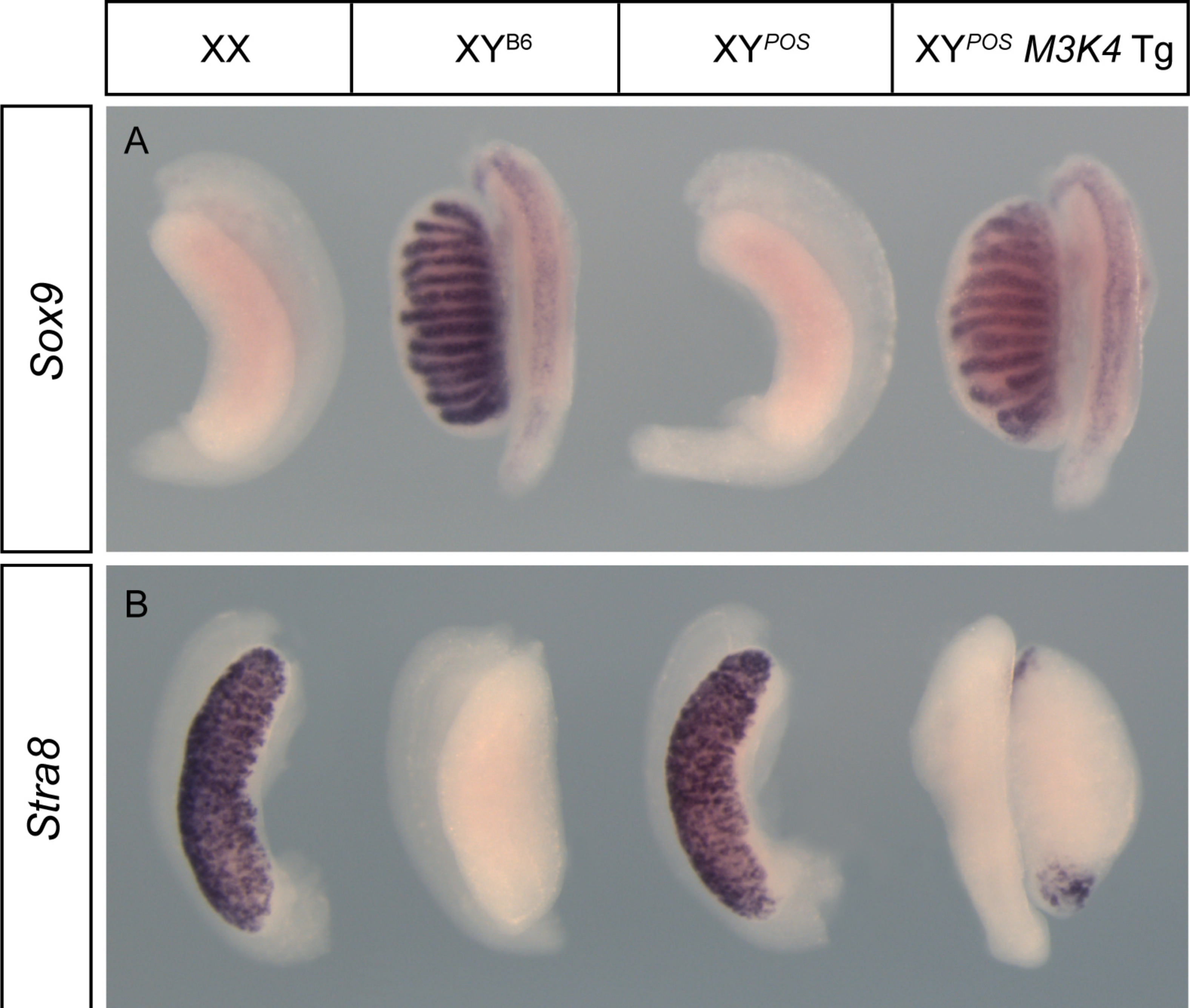

Figure S1

Supplement: Figure S1. Rescue of fetal B6.YPOS gonadal sex reversal by BAC transgenic overexpression of Map3k4. Wholemount in situ hybridisation (WMISH) of (left to right) B6.XX wild-type, B6.XY (XYB6) wildtype, B6.XYPOS and transgenic B6.XYPOS (M3K4 Tg) gonads at 14.5 dpc with Sox9 (A) and Stra8 (B) probes. B6 [file supplementary_figure_1.pdf]

$XY^{B6} +/+$

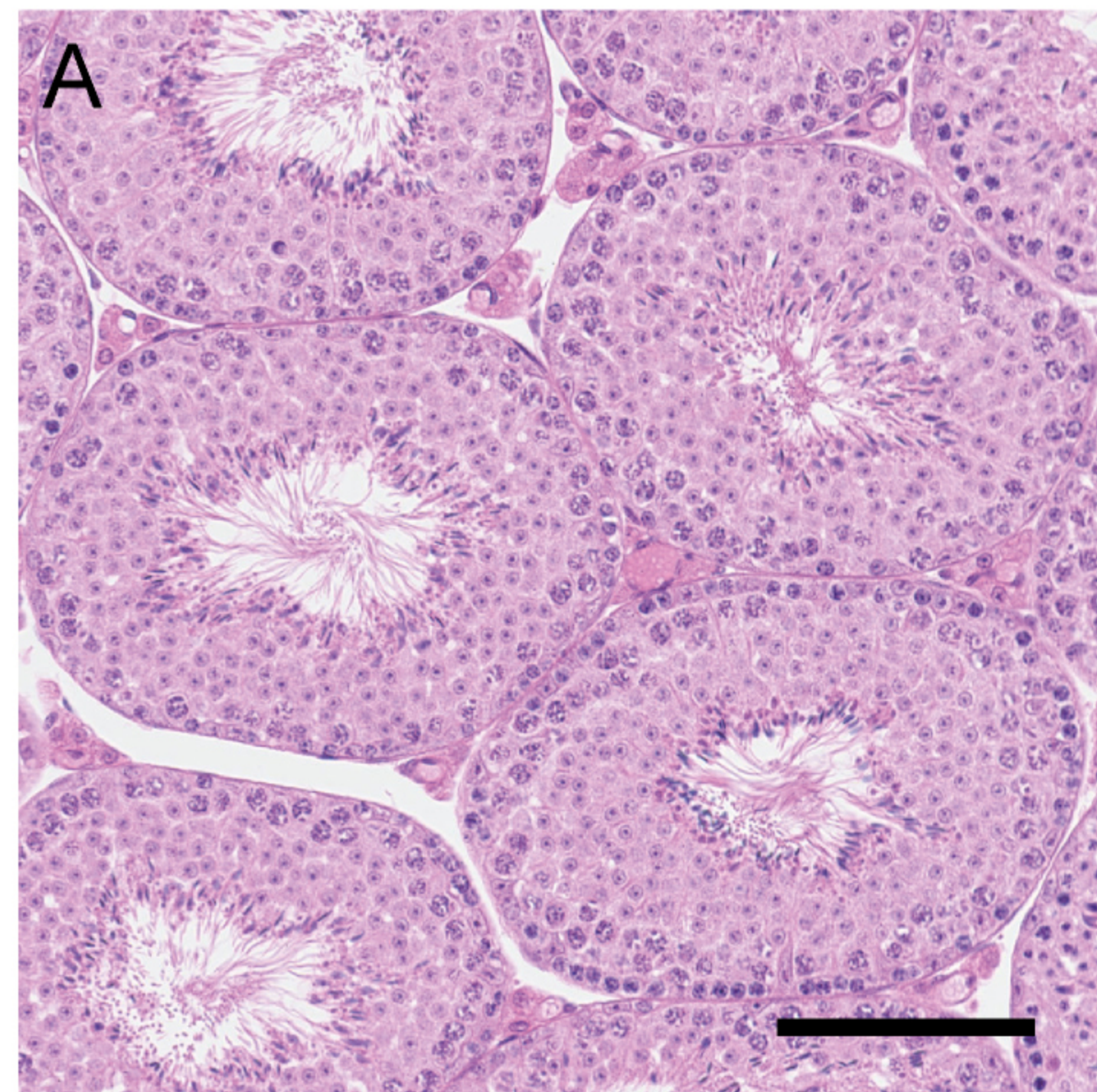

$XY^{POS} +/R$

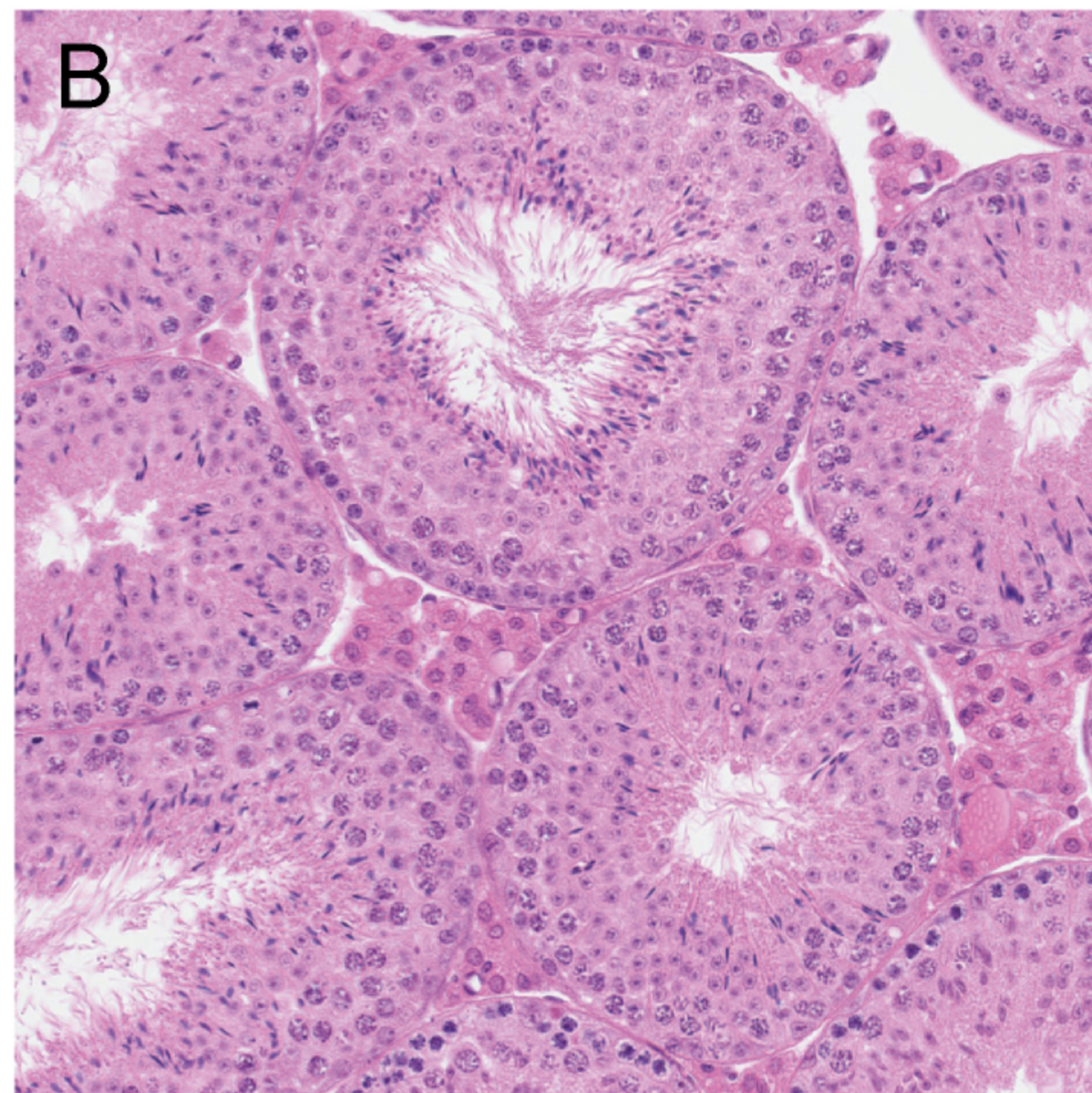

$XY^{B6} G/G +/R$

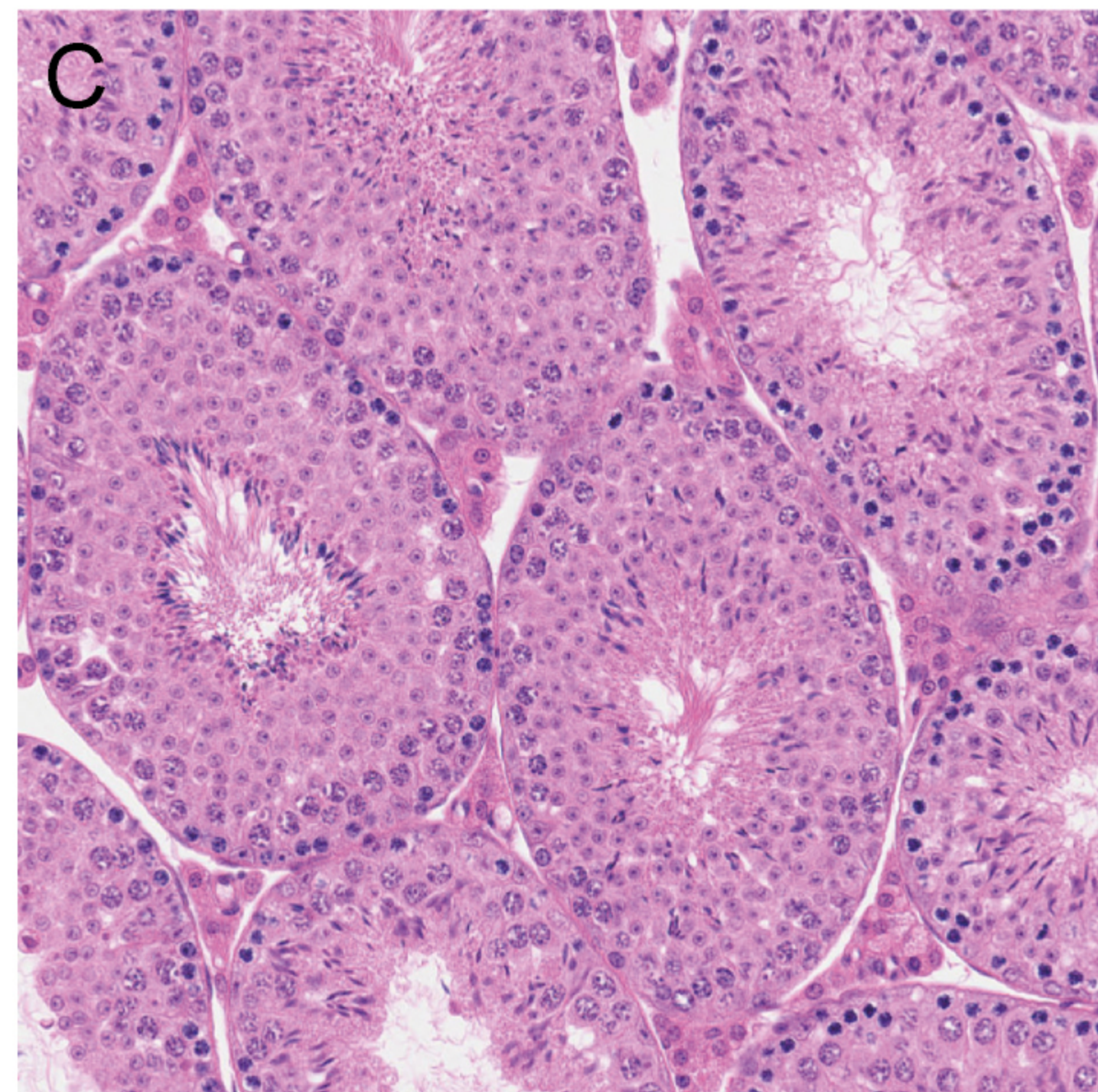

Figure S2

Supplement: Figure S2. Histological examination of adult testis sections. A) control XY (B6.XYB6 +/+), B) B6.XYPOS +/Rspo1 heterozygous (+/R) and C) compound B6.XYB6 homozygous Gadd45g/Gadd45g, heterozygous +/Rspo1 (G/G, +/R) mice reveal no overt differences in anatomy. B6 = C57BL6/J; Scale bar = 100 µm. [file supplementary_figure_2.pdf]

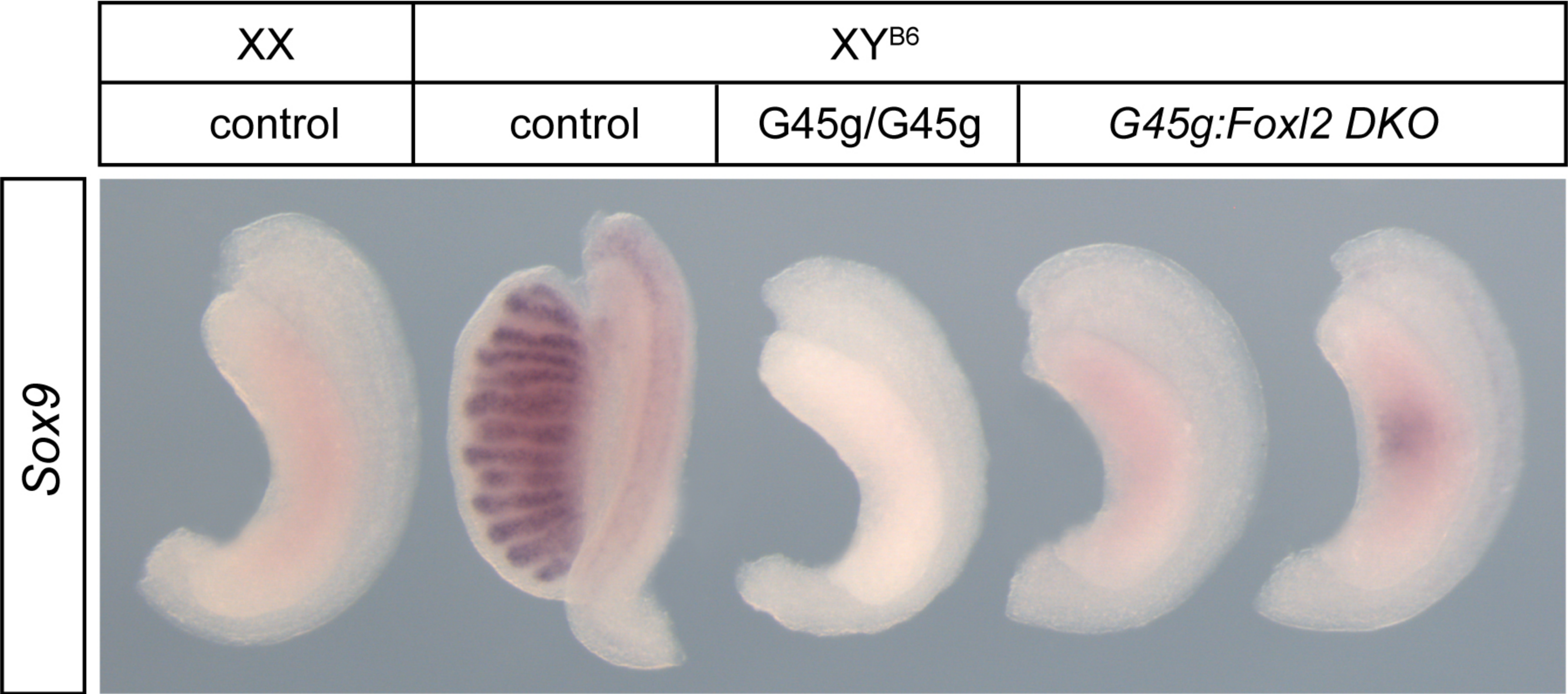

Figure S3

Supplement: Figure S3. XY fetal gonads lacking both Gadd45g and Foxl2 have an ovarian morphology. Comparative Sox9 WMISH analysis of (left to right) B6 control XX and XY, homozygous XY Gadd45g/Gadd45g (G45g/G45g) and doubly homozygous XY Gadd45g/Gadd45g, Foxl2/Foxl2 mutant (G45g:Foxl2 DKO) gonads at 14.5 dpc. O [file supplementary_figure_3.pdf]
